# Investigation of the effectiveness of a classification method based on improved DAE feature extraction for hepatitis C prediction

**DOI:** 10.1038/s41598-024-59785-y

**Published:** 2024-04-21

**Authors:** Lin Zhang, Jixin Wang, Rui Chang, Weigang Wang

**Affiliations:** 1 Zhejiang Hospital of Integrated Traditional Chinese and Western Medicine, Hangzhou, 310003 China; 2 Department of Statistics and Mathematics, Zhejiang Gongshang University, Hangzhou, 310018 China; 3 Department of ICU, Jining No.1 People’s Hospital, Jining, 272100 China

**Keywords:** Hepatitis C, Autoencoder, Denoising autoencoder, Haematological diseases, Mathematics and computing

## Abstract

Hepatitis C, a particularly dangerous form of viral hepatitis caused by hepatitis C virus (HCV) infection, is a major socio-economic and public health problem. Due to the rapid development of deep learning, it has become a common practice to apply deep learning to the healthcare industry to improve the effectiveness and accuracy of disease identification. In order to improve the effectiveness and accuracy of hepatitis C detection, this study proposes an improved denoising autoencoder (IDAE) and applies it to hepatitis C disease detection. Conventional denoising autoencoder introduces random noise at the input layer of the encoder. However, due to the presence of these features, encoders that directly add random noise may mask certain intrinsic properties of the data, making it challenging to learn deeper features. In this study, the problem of data information loss in traditional denoising autoencoding is addressed by incorporating the concept of residual neural networks into an enhanced denoising autoencoder. In our experimental study, we applied this enhanced denoising autoencoder to the open-source Hepatitis C dataset and the results showed significant results in feature extraction. While existing baseline machine learning methods have less than 90% accuracy and integrated algorithms and traditional autoencoders have only 95% correctness, the improved IDAE achieves 99% accuracy in the downstream hepatitis C classification task, which is a 9% improvement over a single algorithm, and a nearly 4% improvement over integrated algorithms and other autoencoders. The above results demonstrate that IDAE can effectively capture key disease features and improve the accuracy of disease prediction in hepatitis C data. This indicates that IDAE has the potential to be widely used in the detection and management of hepatitis C and similar diseases, especially in the development of early warning systems, progression prediction and personalised treatment strategies.

## Introduction

Hepatitis, as an important global public health problem, its early diagnosis and precise treatment are crucial to reduce the disease burden and improve patient prognosis. In recent years, with the rapid growth of biomedical data, how to extract valuable information from massive and complex hepatitis-related data has become a major challenge for medical research. Although traditional machine learning techniques have gradually become the core means of mining the deep value of medical big data, and have made breakthroughs in the accurate diagnosis of diseases^1^, prospective prediction of patient treatment response, and formulation of individualised treatment strategies^2^. However, conventional machine learning methods have revealed a series of inherent limitations when applied to complex medical data like hepatitis C. An et al.^3^ found that conventional machine learning techniques are difficult to effectively mine the non-linear, high-dimensional pathophysiological patterns hidden in highly complex medical data containing multiple clinical indicators and biomarker information. Meanwhile, Rahman et al.^4^ suggested the prevalent category imbalance problem in medical datasets leads traditional machine learning models to be ineffective in dealing with rare and early-stage conditions, and to face significant challenges in terms of robustness and generalisation when coping with situations such as high noise, large amounts of missing data and outliers.

It is in this context that deep learning, as an innovative driver in the field of machine learning, has rapidly penetrated and deepened into medical data analysis in recent years. Traditional models are limited by the complexity, variability and noise of medical data. Therefore, researchers are increasingly adopting deep learning noise reduction techniques to effectively remove medical data noise and extract key features to improve diagnostic accuracy and clinical decision-making efficiency. For example: DnCNN^5^ adopts a residual learning strategy, where the model is dedicated to estimating the residuals of the noisy image relative to its corresponding noiseless original. This innovative approach means that the network only needs to focus on learning the properties of the noisy components, which reduces the difficulty of model training while effectively denoising. Cycle-GAN^6^ proposes the use of a discriminator network for distinguishing the real noise-free image from the image generated by the denoising network, by which the denoising network is forced to generate results closer to the real noise-free image for denoising purposes. RED-CNN^7^ utilises a residual learning structure, where the image features are captured by the encoder and reconstructed inversely in the decoder, and the network focuses on learning the residuals between the noisy image and the noise-free image, thus achieving effective noise removal. FFDNet^8^ dynamically adapts to different noise levels through a noise level-aware deep convolutional network that applies end-to-end learning to remove image noise. While AE, as an unsupervised deep learning framework derived from neural network theory, its variant DAE has likewise been widely used in medical data denoising and disease detection and classification tasks in recent years. For example, Liu et al.^9^ achieved effective extraction of extracted depth features from breast cancer gene expression and CNA data by changing the encoder is realistic dual input denoising autoencoder. Im et al.^10^ combined a noise-reducing autoencoder and a variational autoencoder to denoise the data by introducing random noise and optimising the distribution of hidden variables to learn a robust and interpretive low-dimensional representation of the data during the training process.

However, the application of noise-reducing autoencoders to hepatitis C data has encountered several challenges: firstly, limited by small datasets, noise-reducing autoencoders are prone to simplify the feature overload in a small number of samples, and the deepening of the network may lead to gradient problems and performance degradation due to the high complexity of the features; secondly, the ability of the model to generalise is highly dependent on the type and strength of the added noise; and thirdly, the opacity of the deep learning models limits the intuitive understanding of predictive causal logic, which is critical for medical decision-making. In order to address the above problems and achieve fast convergence of the shallow network while learning more advanced features of the hepatitis C data, we propose to use an improved denoising autoencoder(IDAE), which introduces the concept of ResNet^11^ residual neural network in computer vision for compensating the features that are masked by the data itself by the random noise added to the input. The extracted features are finally used for hepatitis C disease detection. This work is expected to provide a powerful data-driven tool for early diagnosis and individualized treatment of hepatitis, as well as provide lessons and insights for deep learning feature extraction research for other chronic diseases The following are the main contributions of this paper:

- In this paper, machine learning algorithms such as SVM, KNN, Random and various types of autoencoder were used to model and analyze the hepatitis C data and determine the optimal model. It fills the gap in the field in the study of hepatitis C using advanced algorithms. It broadens the depth of knowledge about the complex data characteristics of hepatitis C and promotes the development of advanced algorithmic applications in the study of this disease.
- Applying the concept of residual neural network to DAE enhances the robustness of DAE, improves the stability and reliability of the learnt features, and validates the feature learning capability of IDAE on the hepatitis C dataset. Meanwhile, using ablation experiments, it is demonstrated that IDAE accelerates the convergence of the model due to the inclusion of residual neural networks. The classification effect can also be improved by continuously deepening the neural network depth.
- The enhanced noise-reducing autocoder performs well for feature extraction from the hepatitis C dataset, which may be applied to downstream classification tasks to speed up testing and lower the risk of hepatitis C transmission during the window period, which has some clinical value.

The article is structured as follows: “Related work” describes related work on deep learning in the field of liver, and encoders for medical applications. “Methods” discusses the model used in our work and its structure and included concepts. “Experiments and results” summarizes our experimental findings and observations and evaluates the model from several perspectives to support the model. “Conclusion” summarizes the research in this paper.

## Related work

### Application of machine learning to hepatitis disease detection

In recent years, machine learning (ML) techniques have played an increasingly important role in medical diagnosis and disease management, especially in the field of infectious diseases, such as the diagnosis, prognostic assessment, and selection of therapeutic strategies for Hepatitis C. With the help of Machine Learning techniques, Barakat et al.^12^ developed an intelligent diagnostic system by analysing data from 166 Egyptian chronic hepatitis C (CHC) children’s data, new APRI and FIB-4 cut-offs for predicting fibrosis were identified and predicted using a random forest (RF) model, which showed that RF performed well in fibrosis prediction and staging and was consistent with APRI and FIB-4 metrics, confirming the important role of machine learning in the non-invasive prediction of paediatric hepatic fibrosis.Mostafa et al^13^. used Artificial Neural Networks (ANN), Support Vector Machines (SVM) and RF to analyse and predict liver diseases by dealing with missing data, variable importance ranking and oversampling techniques, and found RF to be the best performing model with an accuracy of over 98%.Similarly, Oladimeji et al.^14^ used Decision Tree (DT), RF, k Nearest Neighbours (KNN), Logistic Regression (LR) and Plain Bayes (NB) combined with SMOTE technique to solve the data imbalance problem to classify the diagnostic tests for Hepatitis C. The performance of the model was evaluated by various metrics and the results showed that RF outperformed the other models, with an AUC-ROC of 0.99 and an accuracy rate of 98.97%. Safdari et al.^15^ investigated the use of machine learning techniques to address data imbalance by employing SMOTE to develop six classification models including SVM, Gaussian NB, DT, RF, LR, and KNN to classify patients with suspected HCV infection, based on the University of California, Irvine HCV dataset. Ultimately, the Random Forest (RF) model stood out in the performance evaluation with an accuracy of 97.29% and an AUC value of 0.998, demonstrating the efficiency of the model in predicting and classifying the stage of HCV infection. Li et al.^16^ developed an artificial intelligence-based two-stage hybrid model combining random forest and logistic regression algorithms to optimise critical thresholds through an artificial bee colony algorithm to automatically classify hepatitis C virus infections with multi-class probabilities, and the Cascade RF-LR (with SMOTE) model was validated by Monte Carlo cross-validation and quantitative metrics comparisons, and was found to be effective at identifying the early onset of HCV and improving the efficacy of treatments

In addition, with the development of medical informatisation, the amount of medical big data is proliferating, which lays a rich data foundation for the expansion of deep learning applications in various fields of medicine, such as diagnostic imaging (CT, MRI, PET, etc.) and electronic medical record analysis. For example, at the forefront of medical image processing research, the U-Net^17^ architecture, as a convolutional neural network model that combines an encoder-decoder structure and cleverly integrates shallow to deep features through hopping connections to achieve high-precision image segmentation, has been widely introduced to medical image segmentation tasks. Although the U-Net architecture itself suffers from high computational cost and overfitting, its simple U-shaped design and excellent performance have led to the emergence of many new network architectures that are similar to its concept, such as Res-UNet^18^, Dense-UNet^19^, which are representative variants. Among them, the encoder part of Res-UNet follows the concept of ResNet and solves the problem of gradient vanishing in deep neural network training by introducing Residual Block, and the decoder part retains the structural features of U-Net by up-sampling layer by layer and merging the features of the corresponding layers of the encoder by skip-joining them to form the decoding process which contains the residual learning mechanism. decoding process that restores the compressed feature map in the encoder to the size of the original input image. From the architectural level, the above methods developed based on the U-Net principle are, to some extent, intrinsically similar to the construction of autoencoder. One of the inspirations for the design concept of the IDAE proposed in this paper is the innovative practice of Res-UNet.

### Autocoder for medical applications

In medical research and practice, raw medical data often contain a certain level of noise due to factors such as equipment accuracy limitations, hardware stability variables, environmental perturbations, and intrinsic physiological variations of the patient, which to a certain extent hinders the accurate acquisition and effective use of key information. In view of this, deep learning technology, as a powerful data processing tool, has been widely used in the field of medical data noise reduction, aiming at improving data quality, refining more accurate feature expression, and ultimately empowering clinical diagnosis and treatment strategy formulation. For example, For example, Asem Khmag^20^ proposed a moment invariant based clustering and Hidden Markov Model (HMM) for preclassification to capture the dependency of additive Gaussian white noise pixels and their neighbouring pixels on the wavelet transform.The HMM also allows the denoising of images by allowing hidden states to be interconnected in order to capture dependencies between coefficients in the transform domain. In addition, Asem Khmag^21^ proposed an innovative adaptive adversarial network algorithm that incorporates noise suppression techniques and adaptively learnt Generative Adversarial Networks (GAN) mechanism, which firstly preprocesses the digital image by combining the image features, and then additionally suppresses the noise using adaptively learnt GAN models to obtain higher visual quality results. In addition, semi-soft thresholding is also used to remove residuals and avoid the phenomenon of “over-smoothing”, and both of the above algorithms can be used in medical image denoising.

However, the most used in medical data is the Denoising Autoencoder (DAE) DAE forces the encoder to learn to restore the original signal from contaminated data, allowing the encoder to learn a more robust and generalised feature representation. With the combination of deep learning and the medical field, DAE is widely used in disease detection. For example, Liu et al.^9^ proposed a design scheme for dual-input unsupervised denoising autocoders (DIUDA), where the design contains two hidden layer constructions in the encoding stage and is equipped with one hidden layer unit in the decoding stage. The DAs model cleverly links the two consecutive encoding layers to jointly process the gene expression data and CNA data derived from the TCGA breast cancer project, effectively realizing the efficient extraction of biomarker features. Experimental evidence confirms that the model can effectively mine and extract biologically meaningful features from genomic data. By utilizing the concept of the AutoEncoder, Chenggang Lyu^22^ suggested a two-stage encoder-decoder based brain tumor sub-region segmentation model. To avoid the overfitting issue, regularization is applied in both stages. On the BraTS 2020 brain tumor dataset, the model’s improvement is also seen. For extremely complex medical data, noise-reducing autoencoders can only handle a single level of random noise and may not be able to adequately extract high-level abstract features. Therefore, the Vincent et al proposed stacked denoising autoencoder^23^ (SDAE). By stacking multiple DAE, SDAE can build deeper network structures, giving the model the ability to learn more complex and abstract feature representations, which can be used to extract important features from medical data. It is currently successfully applied to industrial product detection^24,25^, and medical image detection. Guan et al.^26^ constructed an innovative multi-label learning model based on a stacked denoising autoencoder, which enhances the low-dimensional coding effect produced by the model by optimizing the intrinsic representation learning capability, especially when coping with partially impaired input patterns, in order to capture the low-dimensional coding effect on the noise portion of the input data with robustness. intermediate-level representations that are robust to the noisy portion of the input data, and consequently generating better quality low-dimensional coding. Gu et al.^27^ proposed a variant model of Stacked Denoising Autoencoder (SdA) for molecular typing studies of renal clear cell carcinoma (ccRCC), which in turn aids in the diagnosis of the disease, personalized therapeutic decision-making, and prognostic assessment. This study successfully identified two unique subtypes of ccRCC using five genomic datasets related to renal clear cell carcinoma (KIRC) provided by TCGA. Xu et al.^28^ proposed the use of Stacked Autoencoder (SAE) technology for high-level feature learning for each individual histological dataset and integrated it into the single-layer autoencoder framework in order to obtain more complex fusion representations for fine-grained subtype classification of cancer patients. In addition, other different types of autoencoders^29–31^ have arisen as research in the medical field has deepened. However, the above mentioned methods mainly focus on cancer detection applications, and it is worth noting that the noise-reducing autoencoder has the potential to exacerbate the complexity and challenge of training in the trade-off between noise suppression and effective feature learning, which in turn leads to difficulties in model convergence and is prone to the problem of gradient vanishing or gradient ex plosion, especially in deep neural network architectures. The IDAE proposed in this paper aims to target such convergence difficulties and the phenomenon of gradient vanishing or gradient explosion that may occur in deep neural networks, and its effectiveness is verified on the hepatitis C dataset.

## Methods

### General autoencoder

**Figure 1:**
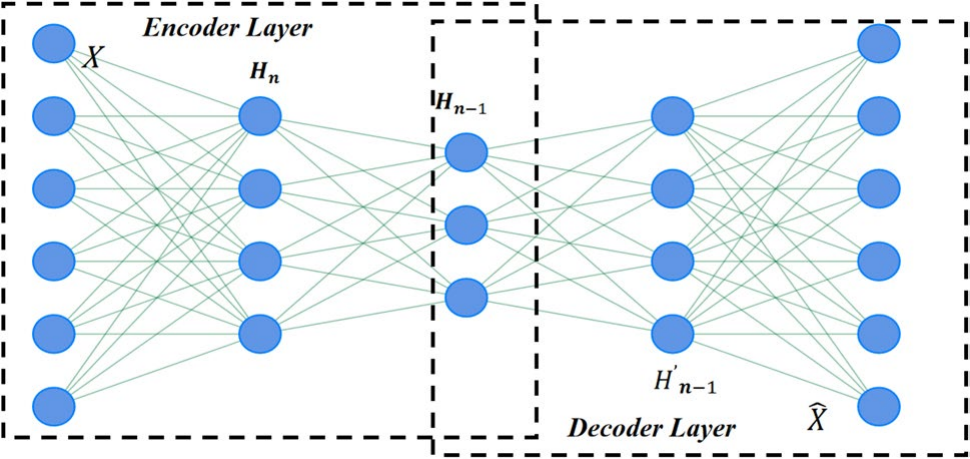
The overall architecture AE model.

Autoencoder(AE) is a special type of neural network model that consists mainly of an encoder and a decoder. The encoder is responsible for transforming the input data into a low-dimensional, dense latent representation, while the decoder tries to restore the original data from this compressed representation. The whole process motivates the model to capture the most essential features in the data, and Fig. 1 illustrates the overall will structure of the autoencoder.

AE does not expect its output to be strictly consistent with the input in its regular operation, but rather seeks to maximally approximate the reconstruction of the input data by implementing an effective data encoding mechanism or implicitly learning the intrinsic representation of the data. Typically, the dimensionality of the output feature vectors generated by the autoencoder will be lower or equal to the dimensionality of the initial input vectors. The model is mostly used for feature extraction in practice, but is also suitable for tasks such as denoising and data dimensionality reduction.The operation of AE can be interpreted as a process of encoding the data from the input layer to the hidden layer, and then decoding the data from the hidden layer to the reconstruction layer, with the following formula for the encoding phase:

1 $$\begin{aligned} {\textbf{H}}={\textbf{g}}\left( {\textbf{W}}_e{\textbf{X}}+{\textbf{b}}_e\right) \end{aligned}$$

Where ${\textbf{X}}={\textbf{R}}^{d\times n}$ is the input of the AE, ${\textbf{g}}$ is an activation function, ${\textbf{H}}$ is the output of the encoder, $\mathbf {W_e}$ is the weight matrix between the encoding and input layers, and $\mathbf {b_e}$ is the node bias of the encoding layer, which is converted to a signal ${\textbf{H}}$, which maps the input data to a low-dimensional hidden space through several hidden layers.

Receiving the output signal ${\textbf{H}}$ from the encoder, the decoder is in charge of decoding the features that were encoded through the activation function g to produce the reconstructed signal ${\textbf{X}}$. The decoder can be viewed as a reconstruction function that strips away extraneous data from the encoder output. The reconstruction function looks like this:

2 $$\begin{aligned} \widehat{{\textbf{X}}}=\mathbf {g^{'}}\left( {\textbf{W}}_d{\textbf{H}}+{\textbf{b}}_d\right) \end{aligned}$$

where ${\textbf{H}}$ is the input, $\widehat{{\textbf{X}}}$ is the decoder’s output, $\mathbf {W_d}$ is the weight matrix of the decoding layer, $\mathbf {b_d}$ is the decoding layer’s node bias, and $\mathbf {g^{'}}$ is the node activation function. The objective of the AE is to obtain the matrix ${\textbf{W}}$ and the bias ${\textbf{b}}$ at the moment the loss function between ${\textbf{X}}$ and $\widehat{{\textbf{X}}}$ is minimized, that is:

3 $$\begin{aligned} \arg \min _{{\textbf{W}},{\textbf{b}}}J\left( {\textbf{W}},{\textbf{b}}\right) \end{aligned}$$

To calculate the separation between ${\textbf{X}}$ and $\widehat{{\textbf{X}}}$, AE typically utilizes the squared error loss function or cross entropy loss function. The loss function of the model is the mean square error or cross entropy of each training session. For the input sample ${\textbf{X}}=\left\{ {\textbf{x}}_i\in {\textbf{R}}^d\right\} _{i=1}^n$ and reconstruction $\widehat{{\textbf{X}}}=\left\{ \widehat{{\textbf{x}}}_ i\in {\textbf{R}}^d\right\} _{i=1}^n$, the algorithm’s precise optimization objective function is as follows:

4 $$\begin{aligned} \left\{ \begin{aligned} J\left( {\textbf{X}},\widehat{{\textbf{X}}}\right)&=\frac{1}{2}\sum _{i=1}^n\Vert \widehat{{\textbf{x}}}_i-{\textbf{x}}_i\Vert _2^2,\\ J\left( {\textbf{X}},\widehat{{\textbf{X}}}\right)&=-\sum _{i=1}^n\left[ {\textbf{x}}_i\log \left( \widehat{{\textbf{x}}}_i\right) +(1-{\textbf{x}}_i)\log \left( 1-\widehat{{\textbf{x}}}_i\right) \right] . \end{aligned} \right. \end{aligned}$$

Typically, during model pre-training, we decide to use AE for feature extraction. We also decide to use multiple AEs cascaded to create stacked AutoEncoders, and we use layer-by-layer greedy training to use the hidden layer output of the previous AE as the input of the next AE for hierarchical feature extraction, which is used as a downstream classification or regression task.

**Figure 2:**
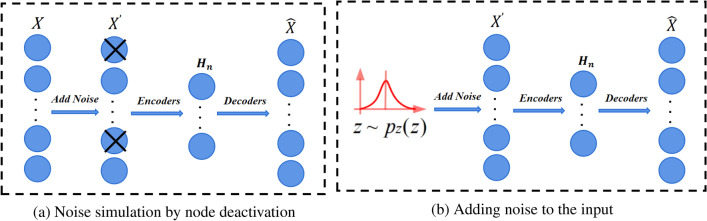
Two DAE architectures, the left figure simulates noise through node deactivation, and the right figure adds noise, such as Gaussian white noise, directly to the input again.

The structure of DAE and AE is similar, the difference is that the features of input data extracted by AE model are easily contaminated by noise.DAE is based on autoencoder, in order to solve the problem of overfitting, some noise is artificially added to the input data to simulate the loss of information, which reduces the dependence of the model on the input features to a certain extent, and makes the learning of the autoencoder robust to a certain extent. There are two common forms of DAE, one as shown in Fig. 2a, where random deactivation is applied as a form of noise injection in the encoder part. Stochastic deactivation usually temporarily shuts down a portion of the neurons during training, which again forces the network to learn a more robust representation of the features. The other, shown in Fig. 2b, adds noise directly in the encoder section, and a common type of noise is normally distributed noise with mean 0 and adjustable standard deviation.

DAE is widely used in medical data, especially in both denoising and feature learning of medical image data: DAEs are able to learn potential low-dimensional feature representations of complex medical data through training, which are usually more interpretive and discriminative than the original data, and help in the classification of diseases, detection of lesions, and tracking of disease progression. However, the complex biology of the hepatitis C virus and individual differences between patients lead to complex nonlinear interrelationships between features, and in order to learn such relationships, it is often necessary to deepen the layers of the encoder, but then introduce the problem of gradient vanishing or gradient explosion, and in the trade-off between noise suppression and effective feature learning, which can exacerbate the complexity and challenge of training, and consequently lead to difficulties in model convergence .

### Improved denoising autoencoder

**Figure 3:**
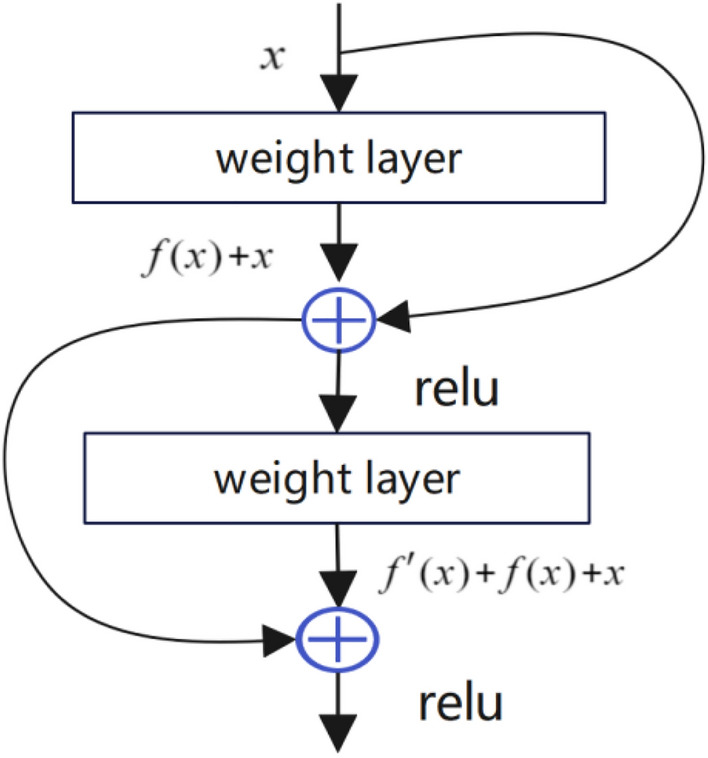
Model structure of the forward propagation layer in residual neural networks.

In this work, we propose an IDAE that incorporates a residual network module. By integrating the residual module and establishing a direct hopping connection between the encoder and the decoder, we effectively construct a straight-through path from the input to the output, solving the problem of gradient disappearance or gradient explosion that occurs in the traditional deep network with the increase in the number of layers, and thus significantly improving the training efficiency and the overall performance of the model. This improvement not only helps to improve the dynamic characteristics of the gradient flow through the deep network, but also helps to simplify the optimization steps, so that the network can converge to the global optimal solution more quickly during the optimization iteration process, which significantly reduces the time required for model convergence. Specifically, compared to general denoising autoencoder, the IDAE proposed in this paper employs a residual neural network, as shown in Fig. 3. It also uses a symmetric encoding and decoding structure, adding the output of each fully connected layer of the encoder directly to the output of the fully connected layer of the corresponding decoder as the input of the fully connected layer of the subsequent decoder.Additionally, a fully connected network is included as a classifier, the model is supervised trained using labeled data, and then the DAE with classification function is obtained by fine-tuning the parameters of the entire network using the inverse algorithm and using the high-level features obtained from the training as inputs to the conventional supervised algorithm.

**Figure 4:**
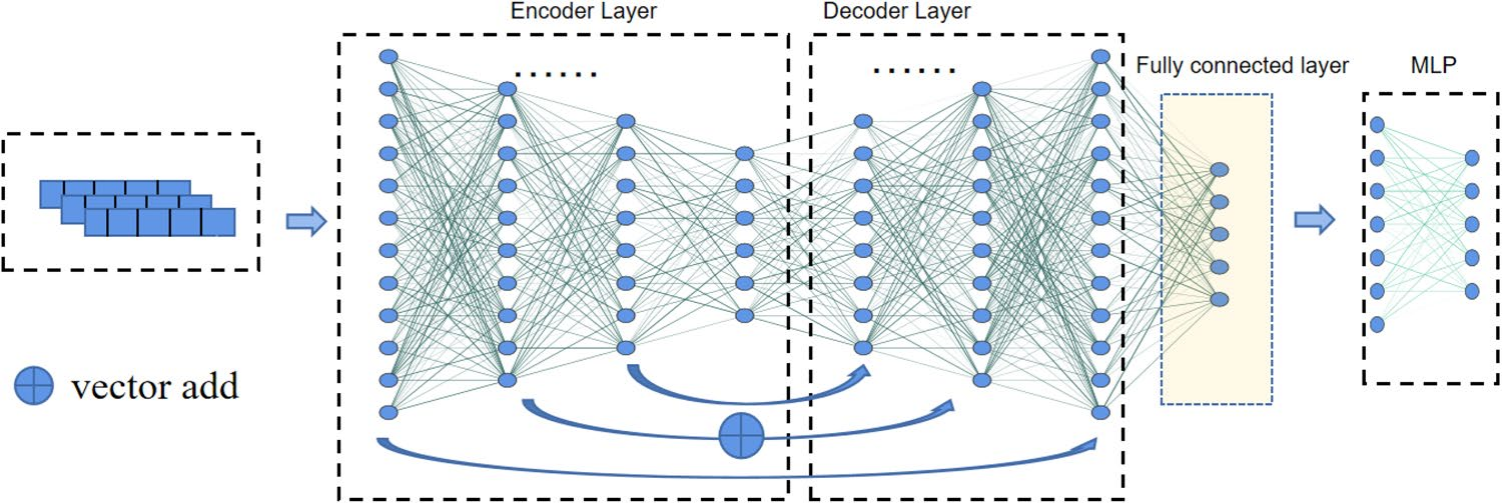
The overall architecture of IDAE.

The structure of the model proposed in this paper is shown in Fig. 4:three fully linked layers are present in both the encoder and the decoder, and a classifier is introduced as a fourth fully connected layer. The residual deities introduce the network connection structure into the model. The corresponding layers of encoders and decoders are realized as layer-hopping connections between them, vectorially summing the inputs of the encoder units of the corresponding layers with the decoder output vectors, and then passing through one fully connected layer and the activation function.

The algorithm itself is sensitive to hyperparameters like degradation rate; too small a degradation rate makes it difficult to improve the performance of the algorithm efficiently, while too large a degradation rate causes the input samples to become seriously distorted and reduce the accuracy of the algorithm. Adding additional degradation processes to the model increases both the training time of the model and the algorithm’s performance. As a result, the enhanced noise reduction dynamic encoder decides to directly inject Gaussian noise rather than simulating the noise with the input layer dropout.

The coding procedure of IDAE is described in the following equation:

5 $$\begin{aligned} {\textbf{H}}_{noise}={\textbf{g}}\left( {\textbf{W}}_e{\textbf{X}} +{\textbf{b}}_e+{\textbf{X}}_{noise}\right) \end{aligned}$$

where ${\textbf{X}}$ is the encoder’s input and consists primarily of the output of the layer before it plus random noise; ${\textbf{X}}_{noise}$ follows a Gaussian distribution; ${\textbf{W}}_e$ and ${\textbf{b}}_e$ are the encoder’s parameter matrices and biases; and ${\textbf{H}}_{noise}$ is the encoder’s output.

The decoding procedure is as follows:

6 $$\begin{aligned} \widehat{{\textbf{X}}}=g\left( ({\textbf{W}}_d{\textbf{H}}+{\textbf{b}}_d )|| ({\textbf{W}}_e{\textbf{X}}+{\textbf{X}}_{noise})\right) \end{aligned}$$

where ${\textbf{W}}_d$ and ${\textbf{b}}_e$ are the parameter matrices and biases of the decoder, $g$ is the relu activation function, $(.||.))$ signifies the splicing of the two vectors, and ${\textbf{b}}_e$ is the output of the decoder.

In this study, the classification model uses the cross-entropy loss function as the cost function, and the parameters are updated similarly to the AE, using the gradient descent method to find the best solution that minimizes the loss function $J\left( W_e,W_d,b_e,b_d\right)$ in order to update the parameter matrix and the bias:

7 $$\begin{aligned} J\left( W_e,W_d,b_e,b_d\right) =\sum _{\textrm{i}}^{\textrm{n}} \left\| X-{\widehat{X}}\right\| _2^2 \end{aligned}$$

8 $$\begin{aligned} \arg \min _{{\textbf{W}},{\textbf{b}}}J\left( {\textbf{W}}_e, {\textbf{W}}_d,{\textbf{b}}_e,{\textbf{b}}_d\right) \end{aligned}$$

Most importantly, the enhanced noise-reducing autoencoder model has the ability to adapt to and capture such complex nonlinear features by increasing the network depth, which is expected to extract more discriminative and interpretive features of high-quality data in the face of complex nonlinear biological interactions, such as those of hepatitis C patients.

## Experiments and results

In this subsection, we evaluate the feature extraction effect of the IDAE by conducting experiments on the Hepatitis C dataset with different configurations to test its generalization ability. We would like to investigate the following two questions:

- How effective is IDAE in classifying the characteristics of hepatitis C ?
- If the depth of the neural network is increased, can IDAE mitigate the gradient explosion or gradient vanishing problem while improving the classification of hepatitis C disease ?
- Does an IDAE of the same depth tend to converge more easily than other encoders on the hepatitis C dataset ?

### Datasets and baselines

Firstly, out of public health importance, Hepatitis C (HCV) is a global public health problem due to the fact that its chronic infection may lead to serious consequences such as cirrhosis and liver cancer, and Hepatitis C is highly insidious, leading to a large number of undiagnosed cases.It is worth noting that despite the wide application of traditional machine learning and deep learning algorithms in the healthcare field, especially in the research of acute conditions such as cancer, however, there is a significant lack of in-depth exploration of chronic infectious diseases, such as hepatitis C. In addition, the complex biological attributes of the hepatitis C virus and the significant individual differences among patients together give rise to the challenge of multilevel nonlinear correlation among features. Therefore, the application of deep learning methods to the hepatitis C dataset is not only an important way to validate the efficacy of such algorithms, but also an urgent research direction that needs to be put into practice to fill the existing research gaps.

The Helmholtz Center for Infection Research, the Institute of Clinical Chemistry at the Medical University of Hannover, and other research organizations provided data on people with hepatitis C, which was used to compile the information in this article. The collection includes demographic data, such as age, as well as test results for blood donors and hepatitis C patients. By examining the dataset, we can see that the primary features are the quantity of different blood components and liver function, and that the only categorical feature in the dataset is gender. Table 1 shows the precise definition of these fields.

**Table 1:** Description of each field in the hepatitis C patient data set.

Type	Field Name	Field Description	Normal value range
Continuous feature	Age	–	F and M
	ALB	Albumin	35–55 g/L
	ALP	Alkaline phosphatase	0–40 U/L
	ALT	Glutamicpyruvic transaminase	M: 5–40 U/L F: 5–35 U/L
	AST	Glutamic oxaloacetic transaminase	0–40 U/L
	BIL	Bilirubin	5.10–19 $\upmu$mol/L
Classification feature	CHE	Serum cholinesterase	4.3–10.5 U/L
	CHOL	Total cholesterol	2.83–5.18 mmol/L
	CREA	Creatinine substance	M: 50–110 $\upmu$mol/L F: 40–100 $\upmu$mol/L
	GGT	Glutamyl transpeptidase	3–50 U/L
	PROT	Total protein	20–80 mg/L
	Sex	–	F and M

This essay investigates the categorisation issue. The Table 2 lists the description and sample size of the five main classification labels. In the next training, in order to address the effect of sample imbalance on the classification effect, the model will be first smote^32^ sampled and then trained using the smote sampled samples. With a sample size of 400 for each classification.

**Table 2:** Description of each field in the hepatitis C patient data set.

Field name	Field description	Sample size	Smote oversampling
0	Qualified blood donors	533	400
1	Confirmed diagnosis of hepatitis C	24	400
2	Confirmed patients with liver fibrosis	21	400
3	Confirmed patients with liver cirrhosis	30	400
0s	Suspected hepatitis patients	7	400

The aim of this paper is to investigate whether IDAE can extract more representative and robust features, and we have chosen a baseline model that includes both traditional machine learning algorithms and various types of autoencoders, which will be described in more detail below:

- SVM: support vector machines are used to achieve optimal classification of data by constructing maximally spaced classification hyperplanes and use kernel functions to deal with nonlinear problems, aiming to seek to identify decision boundaries that maximize spacing in the training data.
- KNN: the K Nearest Neighbors algorithm determines the class or predictive value of a new sample by calculating its distance from each sample in the training set through its K nearest neighbors.
- RF: random forests utilize random feature selection and Bootstrap sampling techniques to construct and combine the prediction results of multiple decision trees to effectively handle classification and regression problems.
- AE: autoencoder is a neural network structure consisting of an encoder and a decoder that learns a compact, low-dimensional feature representation of the data through a autoreconfiguration process of the training data, and is mainly used for data dimensionality reduction, feature extraction, and generative learning tasks.
- DAE: denoising autoencoder is a autoencoder variant that excels at extracting features from noisy inputs, revealing the underlying structure of the data and learning advanced features by reconstructing the noise-added inputs to improve network robustness, and whose robust features have a gainful effect on the downstream tasks, which contributes to improving the model generalization ability.
- SDAE: stacked denoising autoencoder is a multilayer neural network structure consisting of multiple noise-reducing autoencoder layers connected in series, each of which applies noise to the input data during training and learns to reconstruct the undisturbed original features from the noisy data, thus extracting a more abstract and robust feature representation layer by layer.
- DIUDA: the main feature of Dual Input Unsupervised Denoising Autoencoder is that it receives two different types of input data at the same time, and further enhances the generalization ability of the model and the understanding of the intrinsic structure of the data by fusing the two types of inputs for the joint learning and extraction of the feature representation.

### Configurations

In this paper, 80% of the Hepatitis C dataset is used as model training and the remaining 20% is used to test the model. Since the samples are unbalanced, this work is repeated with negative samples to ensure that the samples are balanced. For the autoencoder all methods, the learning rate is initialized to 0.001, the number of layers for both encoder and decoder are set to 3, the number of neurons for encoder is 10, 8, 5, the number of neurons for decoder is 5, 8, 10, and the MLP is initialized to 3 layers with the number of neurons 10, 8, 5, respectively, and furthermore all models are trained until convergence, with a maximum training epoch is 200. The machine learning methods all use the sklearn library, and the hyperparameters use the default parameters of the corresponding algorithms of the sklearn library.

### Model classification performance

To answer the first question, we classified the hepatitis C data after feature extraction using a modified noise-reducing auto-encoder and compared it using traditional machine learning algorithms such as SVM, KNN, and Random Forest with AE, DAE, SDAE, and DIUDA as baseline models. Each experiment was conducted 3 times to mitigate randomness. The average results for each metric are shown in Table 3.From the table, we can make the following observations.

**Table 3:** Results of various algorithmic indicators.entropy.

$IDAE$	0.9970	0.9862	0.9895	0.9843	0.5180
SVM	0.9056	0.9792	0.7913	0.8924	3.0144
KNN	0.8346	0.9217	0.7719	0.8012	6.1164
RF	0.9556	0.9885	0.8939	0.9421	0.9164
AE	0.8856	0.9795	0.7539	0.8521	5.2164
DAE	0.8920	0.9666	0.7987	0.8842	3.2671
SDAE	0.9472	0.9613	0.8928	0.9414	1.0224
DIUDA	0.9562	0.9646	0.9026	0.9512	0.9122

**Figure 5:**
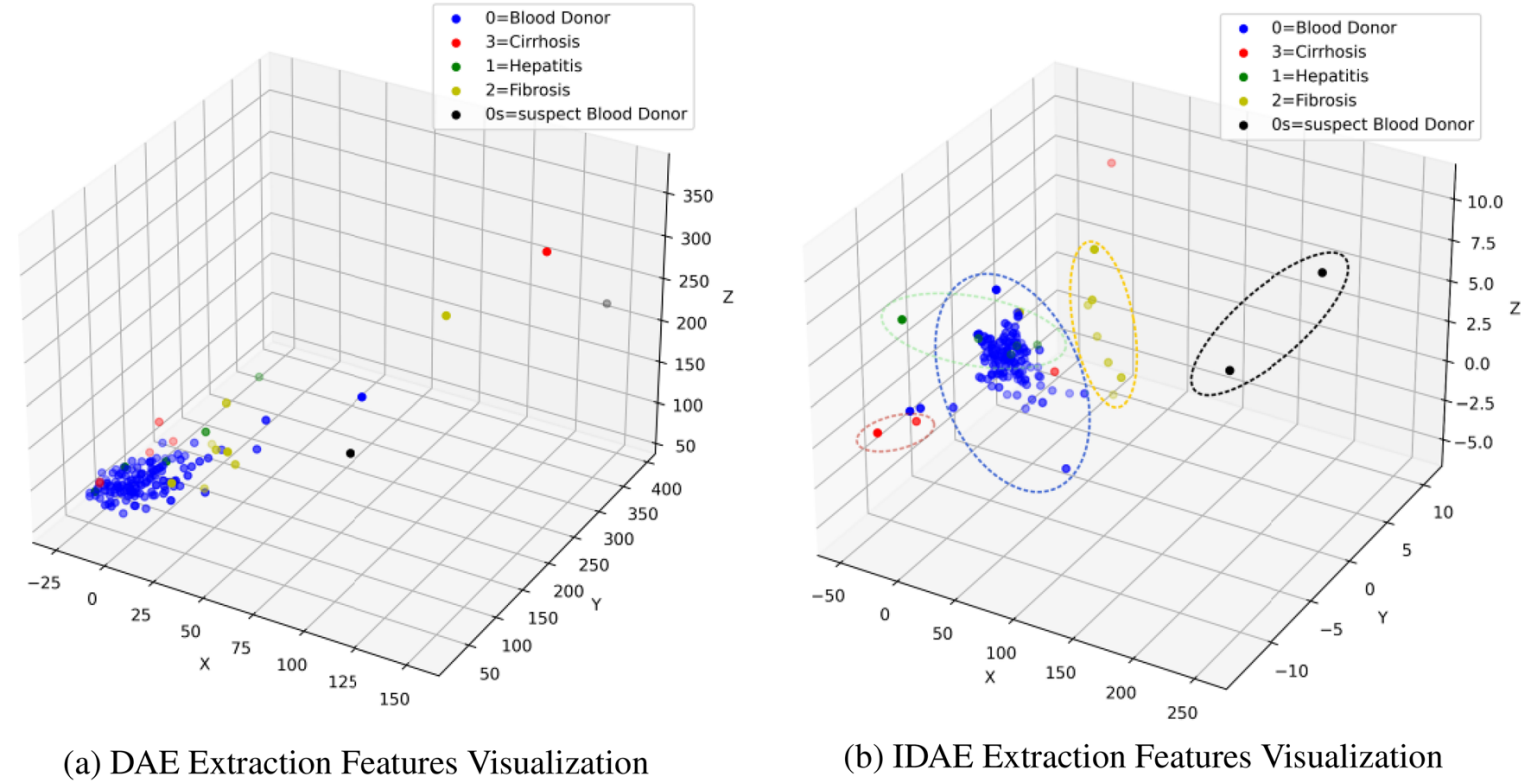
The left figure shows the 3D visualisation of t-SNE with features extracted by DAE, and the right figure shows the 3D visualisation of t-SNE with features extracted by IDAE.

Firstly, the IDAE shows significant improvement on the hepatitis C classification task compared to the machine learning algorithms, and also outperforms almost all machine learning baseline models on all evaluation metrics. These results validate the effectiveness of our proposed improved noise-reducing autoencoder on the hepatitis C dataset. Secondly, IDAE achieves higher accuracy on the hepatitis C dataset compared to the traditional autoencoders such as AE, DAE, SDAE and DIUDA, etc., with numerical improvements of 0.011, 0.013, 0.010, 0.007, respectively. other metrics such as the AUC-ROC and F1 scores, the values are improved by 0.11, 0.10, 0.06,0.04 and 0.13, 0.11, 0.042, 0.032. From Fig. 5, it can be seen that the IDAE shows better clustering effect and class boundary differentiation in the feature representation in 3D space, and both the experimental results and visual analyses verify the advantages of the improved model in classification performance. Both experimental results and visualisation analysis verify the advantages of the improved model in classification performance.

Finally, SVM and RF outperform KNN for classification in the Hepatitis C dataset due to the fact that SVM can handle complex nonlinear relationships through radial basis function (RBF) kernels. The integrated algorithm can also integrate multiple weak learners to indirectly achieve nonlinear classification. KNN, on the other hand, is based on linear measures such as Euclidean distance to construct decision boundaries, which cannot effectively capture and express the essential laws of complex nonlinear data distributions, leading to poor classification results.

In summary, these results demonstrate the superiority of the improved noise-reducing autoencoder in feature extraction of hepatitis C data. It is also indirectly verified by the effect of machine learning that hepatitis C data features may indeed have complex nonlinear relationships.

### Influence of the number of autoencoder layers

**Figure 6:**
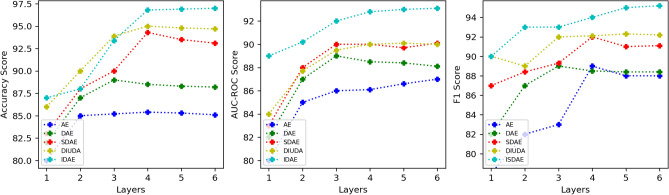
Effects of various types of autoencoders at different depths.

To answer the second question, we analyze in this subsection the performance variation of different autoencoder algorithms at different depths. To perform the experiments in the constrained setting, we used a fixed learning rate of 0.001. The number of neurons in the encoder and decoder was kept constant and the number of layers added to the encoder and decoder was set to {1, 2, 3, 4, 5, 6}. Each experiment was performed 3 times and the average results are shown in Fig. 6, we make the following observations:

Under different layer configurations, the IDAE proposed in this study shows significant advantages over the traditional AE, DAE, SDAE and SDAE in terms of both feature extraction and classification performance. The experimental data show that the deeper the number of layers, the greater the performance improvement, when the number of layers of the encoder reaches 6 layers, the accuracy improvement effect of IDAE is 0.112, 0.103 , 0.041, 0.021 ,the improvement effect of AUC-ROC of IDAE is 0.062, 0.042, 0.034,0.034, and the improvement effect of F1 is 0.054, 0.051, 0.034,0.028 in the order of the encoder.

It is worth noting that conventional autocoders often encounter the challenges of overfitting and gradient vanishing when the network is deepened, resulting in a gradual stabilisation or even a slight decline in their performance on the hepatitis C classification task, which is largely attributed to the excessive complexity and gradient vanishing problems caused by the over-deep network structure, which restrict the model from finding the optimal solution. The improved version of DAE introduces residual neural network, which optimises the information flow between layers and solves the gradient vanishing problem in deep learning by introducing directly connected paths, and balances the model complexity and generalisation ability by flexibly expanding the depth and width of the network. Experimental results show that the improved DAE further improves the classification performance with appropriate increase in network depth, and alleviates the overfitting problem at the same depth. Taken together, the experimental results reveal that the improved DAE does mitigate the risk of overfitting at the same depth as the number of network layers deepens, and also outperforms other autoencoders in various metrics.

### Autoencoder convergence speed

**Figure 7:**
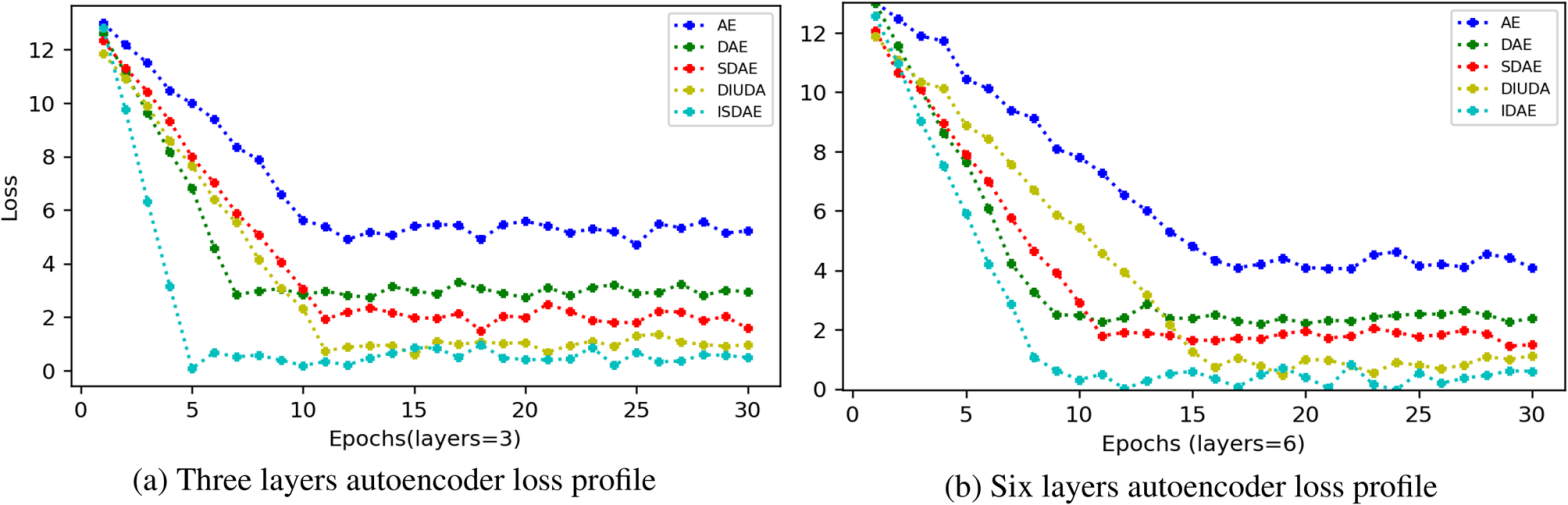
Comparison of model convergence speed for different layers of autoencoders.

To answer the third question, in this subsection we analyse the speed of model convergence for different autoencoder algorithms. The experiments were also performed by setting the number of layers added to the encoder and decoder to {3, 6}, with the same number of neurons in each layer, and performing each experiment three times, with the average results shown in Fig. 7, where we observe the following conclusions: The convergence speed of the IDAE is better than the other autoencoder at different depths again. Especially, the contrast is more obvious at deeper layers. This is due to the fact that the chain rule leads to gradient vanishing and overfitting problems, and its convergence speed will have a decreasing trend; whereas the IDAE adds direct paths between layers by incorporating techniques such as residual connectivity, which allows the signal to bypass the nonlinear transforms of some layers and propagate directly to the later layers. This design effectively mitigates the problem of gradient vanishing as the depth of the network increases, allowing the network to maintain a high gradient flow rate during training, and still maintain a fast convergence speed even when the depth increases. In summary, when dealing with complex and high-dimensional data such as hepatitis C-related data, the IDAE is able to learn and extract features better by continuously increasing the depth energy, which improves the model training efficiency and overall performance.

## Conclusion

This study is dedicated to exploring the potential of machine learning methods in the early diagnosis of hepatitis C. A DAE model IDAE containing a residual neural network structure is innovatively constructed, aiming to alleviate the overfitting problem caused by the random noise in training, and thus to improve the generalisation performance of the model. In addition, by utilising the residual connection design, the IDAE effectively enhances the training efficiency while strengthening the representation learning capability. The research team applied this improved model to the hepatitis C related dataset for unsupervised feature learning, and the experimental results show that compared with the conventional denoising autoencoder and machine learning methods, the model achieves significant improvement in all kinds of evaluation indexes of the hepatitis C dataset. However, in real hepatitis C clinical testing, the recall rate is particularly important because it reflects whether the testing method misses the real cases. Especially in infectious diseases such as hepatitis, a high recall rate can maximise the detection of infected individuals and prevent the spread of the disease. However, despite the excellent performance of IDAE in terms of recall, it is still not possible to completely circumvent the possibility that negative test results may be underdiagnosed, which is an important limitation of the current study. Future research directions are expected to extend IDAE to more feature learning tasks within the healthcare domain, while continued attention and efforts are needed to address the limitations of such models in clinical applications. Looking ahead, given the advantages demonstrated by the improved noise-reducing autoencoder on the Hepatitis C dataset and its potential in medical data research, we believe that its extension to other feature learning tasks within the healthcare domain has positive application prospects. In particular, in the current context of medical data analysis, which is placing more and more emphasis on unsupervised learning techniques, further exploring the applicability of autoencoder techniques in different disease diagnosis, biomarker identification and pathological state classification is undoubtedly a direction worthy of attention and expansion in subsequent research.
